# Metal Matrix Composite in Heat Sink Application: Reinforcement, Processing, and Properties

**DOI:** 10.3390/ma14216257

**Published:** 2021-10-21

**Authors:** Mirza Murtuza Ali Baig, Syed Fida Hassan, Nouari Saheb, Faheemuddin Patel

**Affiliations:** 1 Department of Mechanical Engineering, King Fahd University of Petroleum and Minerals, Dhahran 31261, Saudi Arabia; mmurtuza@kfupm.edu.sa (M.M.A.B.); nouari@kfupm.edu.sa (N.S.); faheemmp@kfupm.edu.sa (F.P.); 2 Interdisciplinary Research Center for Advanced Materials, King Fahd University of Petroleum and Minerals, Dhahran 31261, Saudi Arabia

**Keywords:** metal matrix composite, heat-sink, aluminum matrix composite, reinforcement

## Abstract

Heat sinks are commonly used for cooling electronic devices and high-power electrical systems. The ever-increasing performance of electronic systems together with miniaturization calls for better heat dissipation. Therefore, the heat sink materials should not only have high thermal conductivities, low densities, and cost, but also have coefficients of thermal expansion matching to those of semiconductor chips and ceramic substrates. As traditional materials fail to meet these requirements, new composite materials have been developed with a major focus on metal matrix composites (MMCs). MMCs can be tailored to obtain the desired combination of properties by selecting proper metallic matrix and optimizing the size and type, volume fraction, and distribution pattern of the reinforcements. Hence, the current review comprehensively summarizes different studies on enhancing the thermal performance of metallic matrices using several types of reinforcements and their combinations to produce composites. Special attention is paid to the types of commonly used metallic matrices and reinforcements, processing techniques adopted, and the effects of each of these reinforcements (and their combinations) on the thermal properties of the developed composite. Focus is also placed on highlighting the significance of interfacial bonding in achieving optimum thermal performance and the techniques to improve interfacial bonding.

## 1. Introduction

Heat sinks are commonly used for cooling electronic devices and high-power electrical systems [1]. Chingulpitak et al. [2] and Ahmed et al. [3] defined heat sink as “a type of heat exchanger used as a cooling system in electronic component”. Pawar et al. [4] consider heat sink as an “environment or object that absorbs and dissipates heat from another object using thermal contact (either direct or radiant)”. The advantages of the heat sink are low initial cost, simple installation, and a reliable manufacturing process [2,5]. They are widely used in cooling of electronic equipment and/or components including microprocessors, power modules, lasers, light-emitting diodes (LEDs), plasma and liquid crystal displays (LCDs and thermoelectric coolers (TECs) [4,6,7].

Microelectronics systems find wide application in today’s world, ranging from digital watches to supercomputers [8]. The incessant growth in microelectronic systems has been driven by an insatiable quest for ‘faster-smaller-cheaper’ devices [9,10]. As the speed and operating frequency of the chip increases, the power dissipation goes up [3,8]. A reduction in ‘interconnection-delays’ by densely packing these chips and also hundreds of millions of transistors on a very small area on each chip enhances the performance further [8]. This comes with a penalty of an increase in the power density at the chip and module levels [8,11,12,13,14,15,16,17]. Therefore, the speed increases and the volume reduces at the expense of heat generation [3,6,8,14,15,16,18]. The temperature of the device will rise if the heat is not dissipated at a rate greater than the generation rate. Consequently, the effective life of the component is reduced [3,4,6,11,14,19,20]. In total, 55% of microelectronics failure are reported to be due to higher operating temperatures [1,11,19]. The effective containment of the operating temperature within the design limits can ensure a longer service life and reliable performance [4,12]. Therefore, the demand for improved thermal management solutions in microelectronic packaging has received intense research focus.

**Figure 1:**
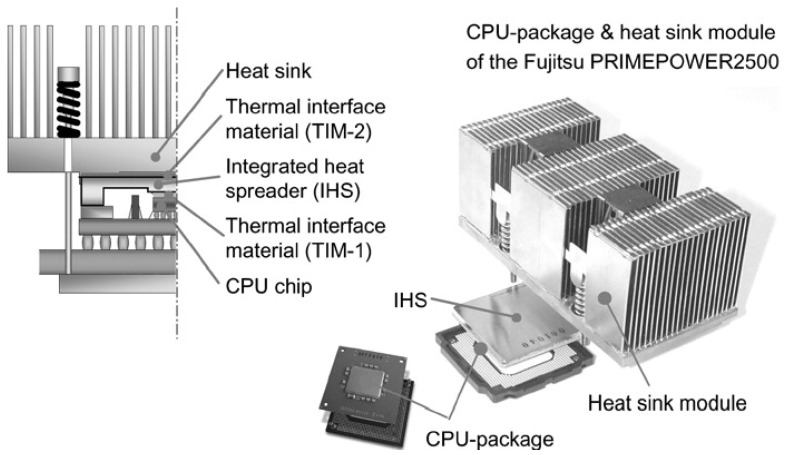
A typical structure of the CPU package and heat sink module [21].

Figure 1 shows a schematic arrangement of the high-performance processor package. The package consists of an integrated heat spreader that is attached adhesively or soldered to the chip using a thermal interface material. The heat spreader spreads the heat from the chip to a wider area heat sink through a thermal interface material. Finally, the heat is dissipated from the heat sink fins to the surroundings [21].

The electronic systems have been continuously evolving following two important trends, namely ‘enhanced performance’ and ‘miniaturization’ [12,22]. The enhancement in performance is achieved at the expense of higher power densities, leading to a higher heat generation rate. To dissipate heat at a greater rate, the heat transfer area of the heat sink may be increased [23]. However, the resulting increase in size and weight is against the miniaturization trend [23,24] and can induce mechanical stresses in the attached components [12,23,24]. Moreover, the end cost may be higher [23]. Therefore, the heat sink materials should not only have high thermal conductivities [3,7,11,12,21] but also be lighter and cheaper [4,7,12,15]. Since the semiconductor chips and ceramic substrate have low coefficients of thermal expansion (i.e., between 3 × 10^−6^ K^−1^ and 7 × 10^−6^ K^−1^) [11], heat sinks must also match those [6,7,11,21]. Traditionally, Cu, Al, Cu-Mo, and Cu-W blends; Cu-Mo-Cu and Cu-Invar (64Fe-36Ni) laminates; and Invar and Kovar (53Fe-29Ni-17Co) alloys have been used as heat sink materials [15,25]. Al and Cu have an unacceptably high coefficient of thermal expansion, which induces thermal stresses, leading to brittle fracture of ceramic substrates. Tungsten and Molybdenum have high densities while Invar and Kovar alloys have poor thermal conductivity and high cost. As traditional materials fail to meet all the requirements, new composite materials have been developed, with a major focus on metal matrix composites. Hence, the focus of this review paper is to comprehensively summarize the types of commonly used reinforcements, processing techniques adopted, and the effects of each of these reinforcements (and their combinations) on the thermal properties of the developed composite.

## 2. Metal Matrix Composites

**Figure 2:**
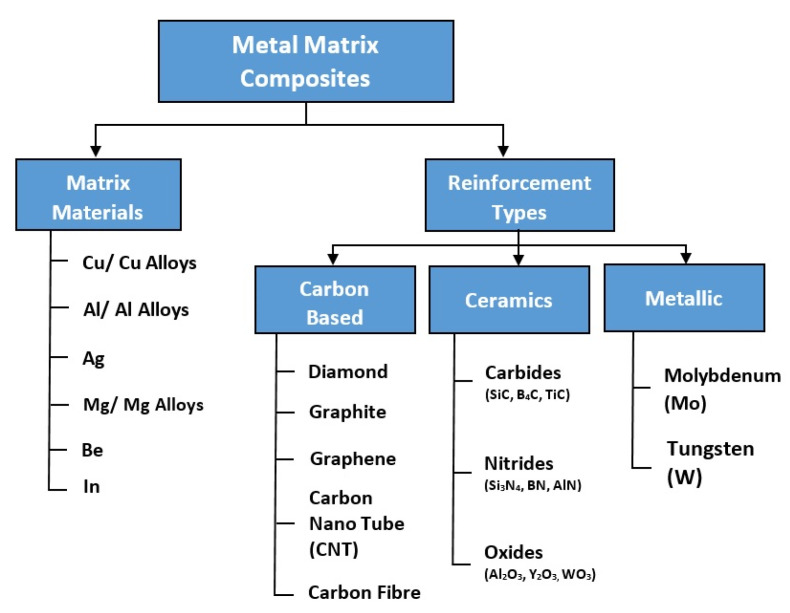
Different types of matrix materials and reinforcements used for heat sink applications.

Metal matrix composites (MMCs) are a type of composite in which, typically, the ceramic reinforcements are embedded in a metallic or alloy matrices [22,26,27,28]. MMCs can be tailored to combine metallic properties (high thermal conductivity, small density, toughness, ductility) with ceramic characteristics (low coefficient of thermal expansion, high strength and modulus), making them the most appropriate candidate as a heat sink material [11,22,25,26,27,28,29,30,31,32,33,34]. The desired combination of thermal conductivity and coefficient of thermal expansion can be obtained by optimizing the size and type, volume fraction, and distribution pattern of the reinforcements [31,34,35].The different types of matrix materials and reinforcements used for heat sink applications are presented in Figure 2.

### 2.1. Copper Matrix Composites

Copper is the most widely used matrix material for heat sink applications due to its high thermal conductivity (400 Wm^−1^K^−1^), high melting temperature, and good weldability [36,37,38,39,40,41,42,43,44,45]. To overcome the high coefficient of thermal expansion of copper, different reinforcements have been incorporated in the copper matrix, namely diamond particles [36,38,39,40,41,44,45,46,47,48,49,50,51,52,53,54,55,56,57,58,59,60,61,62,63,64,65,66,67], graphite (particles, fibers, or foam) [44,68,69,70], carbon fibers (CFs) [39,54,71,72,73], carbon nanotubes (CNTs) [74,75], SiC (particles or fibers) [37,71,76,77], tungsten (particles, fibers, or wires) [42,43,71,78,79], molybdenum particles [80], and a hybrid of Y_2_O_3_ and WO_3_ particles [81].

The achievement of desired thermal properties from the copper matrix composite is determined by the interface between the matrix and the reinforcement [38,46,58,67,82,83,84]. The formation of a weak interfacial bond due to the non-wettability of liquid copper and absence of chemical reactivity with carbon-based reinforcements (diamonds, carbon fibers, and graphite) results in high thermal resistance at the interface. Consequently, the thermal conductivity of such composites is low [38,40,41,44,50,53,54,55,56,57,58,59,60,61,62,63,64,65,67,70,76,84,85,86,87,88,89,90,91]. Two approaches have been adopted in the literature to improve interfacial bonding: alloying copper matrix and coating reinforcements with carbide-forming elements.

#### 2.1.1. Diamond Reinforcements

**Figure 3:**
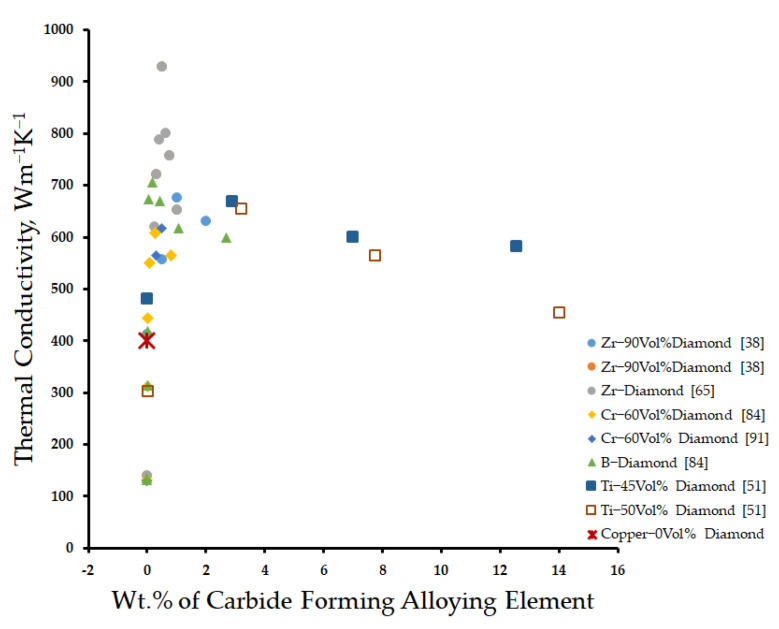
Effect of the content of carbide-forming alloying element in the copper matrix on the thermal conductivity of the composite.

Diamond-reinforced copper matrix composites have attracted the most interest of researchers due to their high thermal conductivity (up to 2200 Wm^−1^K^−1^) [36,41]) and low coefficient of thermal expansion (2.3 × 10^−6^ K^−1^ [41]). To take full advantage of its excellent thermal properties, the copper matrix was primarily alloyed with carbide-forming elements, such as Zr, Cr, B, and Ti. The thermal conductivity of the composite first increases and then decreases with an increasing content of Zr, Cr, B, and Ti as shown in Figure 3. He et al. [38] reported a maximum thermal conductivity of 677 Wm^−1^K^−1^ at 1 wt% Zr. Li et al. [65] and Wang et al. [46] reported a maximum thermal conductivity of 930 Wm^−1^K^−1^ at 0.5 wt% Zr. Bai et al. [62] found that the thermal conductivity of composite approaches 660 Wm^−1^K^−1^ at 5 wt% B. Weber et al. [84] reported maximum thermal conductivities of 600 and 700 Wm^−1^K^−1^ at 0.005 at% and 2.5 at% Cr and B, respectively. Che et al. [54] obtained a maximum thermal conductivity of 670 Wm^−1^K^−1^ by alloying the matrix with 3 vol% Ti. It was observed that the concentration of alloying elements should be optimized to achieve the maximum value of thermal conductivity as shown in Figure 3. At lower concentrations, inadequate interfacial bonding due to the small size of interfacial carbides results in inferior thermal conductivity. At higher concentrations, the thermal resistance of the interfacial carbide adversely affects the thermal conductivity of the composite [38,46,54,65,84]. Moreover, the excess alloying elements will remain in the matrix unreacted and deteriorate the thermal conductivity of the composite [40,53,54,60,64,84].

**Figure 4:**
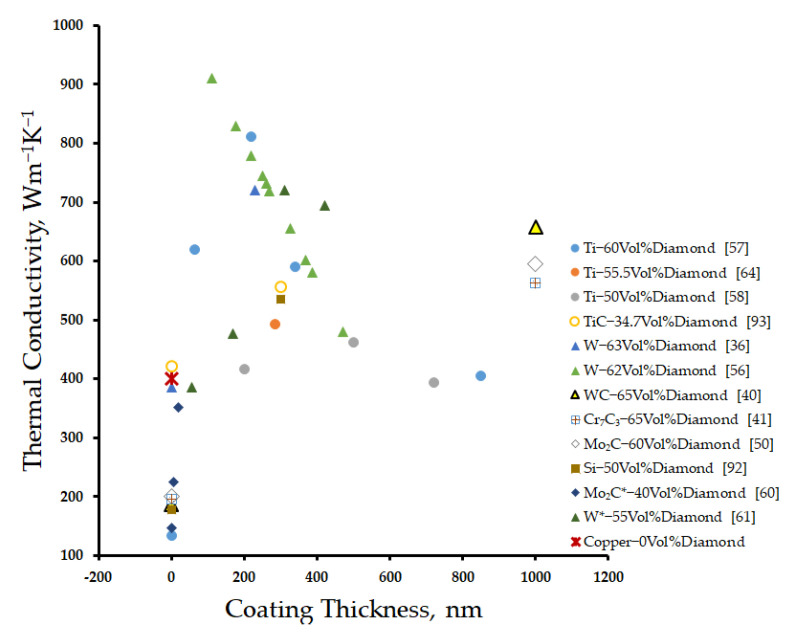
Effect of the carbide coating thickness on the diamond surface on the thermal conductivity of copper matrix composites.

The secondary approach to improve the interfacial bond between diamond particles and the copper matrix is the diamond surface modification. The carbide-forming elements, such as B, Ti, W, Cr, Mo, or Si, are deposited on the surface of diamond by magnetron sputtering [45,49,60,63], the molten salt method [40,41,48,53,67], the diffusion method [36,58,59], vacuum evaporation deposition [91], and electroless chemical deposition [52]. This coating serves as an intermediate layer that not only strengthens the interface between diamond and copper but also mitigates the degree of graphitization of diamond particles at elevated temperatures [53]. Figure 4 not only shows that the thermal conductivity improves with the application such coatings, but also that the larger thicknesses of those coatings can have an adverse effect. A thermal conductivity of 300 Wm^−1^K^−1^ for copper/diamond composite reinforced with (1.9 µm thick) Cu-0.5B-coated diamond particles was reported [45]. Maximum thermal conductivities of 811 [60] and 493 Wm^−1^K^−1^ [67] were reported for copper matrix composites reinforced with diamond particles with 220- and 285-nm-thick Ti coating, respectively. Abyzov et al. [36,58,59] observed that the thermal conductivity of the copper/diamond composite increases from 500 Wm^−1^K^−1^ at 500-nm-thick tungsten coating on diamond reinforcement to 900 Wm^−1^K^−1^ with the reduction in the coating thickness to 100 nm. Kang et al. [40] synthesized a composite yielding a high thermal conductivity of 658 Wm^−1^K^−1^ by applying 1-μm-thick WC coating on the diamond particulate reinforcement. Some researchers have reported thermal conductivities of 562 [41] and 596 Wm^−1^K^−1^ [53] accompanied with coefficients of thermal expansions of 7.8 × 10^−6^ K^−1^ and 7.15 × 10^−6^ K^−1^ with Cr_7_C_3_- and Mo_2_C-coated diamond particulate-reinforced copper matrix composites. Zhu et al. [92] reinforced Si-coated diamond particles into copper matrix to obtain a thermal conductivity of 535 Wm^−1^K^−1^. Cho et al. [93] reinforced TiC-coated diamond particles into copper matrix to obtain a thermal conductivity of 557 Wm^−1^K^−1^. Chang et al. [49] demonstrated that though the intermediate carbide layer can potentially improve the interfacial thermal conductance, the large thickness and low crystallinity of the intermediate layer will have an adverse effect on the thermal conductivity. The increase in thickness from 10 to 20 nm of the intermediate TiC layer deposited on a diamond substrate at room temperature was found to reduce the interfacial thermal conductance from 29 to 19 MW/(m^2^·K). On the other hand, an increase in the coating deposition temperature (10 nm thickness) to 873 K increased the interfacial thermal conductance from 29 to 85 MW/(m^2^·K) due to the transformation of the intermediate TiC layer from an amorphous to crystalline state.

Alternatively, some studies [61] have combined both the techniques by alloying copper matrix with Ti and modifying the diamond surface with a thin Ti coating. The particle composite system used to homogeneously blend Cu-Ti powders caused the finer Ti particles to attach to the coarse Cu powders. The formation of the diamond/TiC/Ti/CuTi/Cu structure at the interface resulted in a thermal conductivity of 630 Wm^−1^K^−1^. In a similar study involving alloying matrix and coating diamond with Cr, a thermal conductivity of 810 Wm^−1^K^−1^ was achieved [91]. In an attempt to avoid the usage of carbide-forming elements, diamond particles were coated with copper nanoparticles [39], but the maximum thermal conductivity that could be achieved was 460 Wm^−1^K^−1^. Some researchers have adopted a new technique of applying a dual layer coating on diamond particles with W [48,64] or Mo [63] as the inner layer and Cu as the outer layer. The resulting thermal conductivities were reported as 721, 661, and 351 Wm^−1^K^−1^, respectively. The advantages of this technique are a uniform distribution of diamond particles, lowering of the sintering temperature, and a very strong interfacial bond.

#### 2.1.2. Graphite Reinforcements

High thermal conductivity (˃900 Wm^−1^K^−1^), negative coefficient of thermal expansion (−1.45 × 10^−6^ K^−1^), and low cost have made graphite fibers a potential reinforcement. The enhanced machinability of the composite is an additional advantage [70]. To improve interfacial bonding, they were coated with Cr. The inplane thermal conductivities and coefficients of thermal expansion were reported to range from 380–412 Wm^−1^K^−1^ and 6.1–9.4 × 10^−6^ K^−1^ [70]. In another study [44], the incorporation of graphite flakes resulted in a maximum thermal conductivity of 560 Wm^−1^K^−1^ perpendicular to the pressing direction. To obtain isotropic properties, W-coated graphite particles were used as reinforcement, but the resulting thermal conductivity reported was just 158 Wm^−1^K^−1^ [69]. A composite presenting a combination of bulk thermal conductivity and coefficient of thermal expansion (i.e., around 342 Wm^−1^K^−1^ and 7.4 × 10^−6^ K^−1^, respectively) suitable for heat sink application was obtained by infiltrating liquid copper into graphite foam [94].

#### 2.1.3. Carbon Fiber Reinforcements

Carbon fibers have a thermal conductivity as high as 900 W/mK and a negative coefficient of thermal expansion (−0.6 × 10^−6^ K^−1^) along the fiber orientation. They were used as reinforcements to synthesize copper matrix composites for thermal management applications. Thermal conductivities of 220 and 120 Wm^−1^K^−1^ of unidirectional composites in the longitudinal and transverse direction, respectively, were reported by Korab et al. [72]. In a similar study [39], optimum thermal conductivities of 330 and 160 Wm^−1^K^−1^ in the longitudinal and transverse directions, respectively, were reported by incorporating 35% volume fraction pitch-type carbon fibers (K6371T) in the copper matrix. In order to overcome anisotropy in properties, cross-ply composites were fabricated, but the thermal conductivities obtained in the in-plane and transverse directions (i.e., 150 and 50 Wm^−1^K^−1^, respectively) were below 300 Wm^−1^K^−1^ [95]. Novel hydrothermal sintering successfully yielded an approximate isotropic thermal conductivity of 300 Wm^−1^K^−1^ with 40% volume fraction of copper-coated carbon fibers [73].

#### 2.1.4. Carbon Nanotubes (CNTs)

CNTs possess outstanding thermal conductivity (3000–6000 Wm^−1^K^−1^) with an extraordinarily low coefficient of thermal expansion (0 × 10^−6^ K^−1^), which can be exploited to achieve enhanced thermal performance. Unfortunately, retaining the thermal conductivity of matrix metal while incorporating CNTs has been a great challenge due to the inhomogeneous dispersion of CNTs. The formation of such clusters deteriorates the thermal conductivity of the composite [74]. A composite exhibiting a thermal conductivity and a coefficient of thermal expansion of 395 Wm^−1^K^−1^ and 5 × 10^−6^ K^−1^, respectively, was successfully fabricated by filling copper in the pores of the macroscopic CNT [75].

#### 2.1.5. Graphene

Graphene possesses outstanding in-plane thermal conductivity in the range of 1000–5300 Wm^−1^K^−1^, and a through-plane thermal conductivity in the range of 5–20 Wm^−1^K^−1^. Moreover, it possesses a negative coefficient of thermal expansion of −1.28 × 10^−6^ to −8 × 10^−6^ K^−1^ [96,97]. Though such outstanding properties make it an ideal reinforcement for MMCs intended for heat sink applications, its proper alignment is essential for an enhancement of its thermal performance. A thermal conductivity of 396 Wm^−1^K^−1^ was reported by reinforcing copper matrix with 0.3 wt% graphene by electrostatic self-assembly and powder metallurgy [98]. Chu et al. [96,97] developed an effective method to obtain copper matrix composites with highly aligned graphene reinforcements by using a vacuum filtration process followed by spark plasma sintering (SPS). Consequently, a copper matrix composite with 30 vol % graphene nanosheets yielded an in-plane thermal conductivity of 458 Wm^−1^K^−1^ along with a low through-plane coefficient of thermal expansion of 6.2 × 10^−6^ K^−1^ [97]. In another study [96], 35 vol% graphene nano-platelet reinforcement lead to a higher in-plane thermal conductivity of 525 Wm^−1^K^−1^. Graphene nano-sheets (1 wt%) when added to Cu/WC-TiC-Co composite improved its thermal conductivity from 190 Wm^−1^K^−1^ to 350 Wm^−1^K^−1^ [99]. The powder mix was coated with copper to ensure good interfacial bonding.

#### 2.1.6. Silicon Carbide (SiC)

SiC possess a thermal conductivity and coefficient of thermal expansion of 200–300 Wm^−1^K^−1^and 4.5 × 10^−6^ K^−1^, respectively. Only a few researchers have focused on investigating the appropriateness of using SiC as a heat sink material. A maximum achieved thermal conductivity of 400 Wm^−1^K^−1^was reported for a composite with 40% volume fraction SiC reinforcements [37]. Since SiC is unstable in copper at high temperatures, it is generally coated with W, Cr [76], and Mo [77]. A thermal conductivity of 306 Wm^−1^K^−1^ with a coefficient of thermal expansion of 11 × 10^−6^ K^−1^ was reported with Mo-coated SiC particle inclusion in copper matrix [77].

#### 2.1.7. Metal Particles

Tungsten (W) is specifically used as a reinforcement in Cu [42,43,78,79] or CuCrZr [36,43,71,100] matrix for heat sinks subjected to high heat flux. Since the composite is subjected to elevated temperatures, it must possess structural, mechanical, and thermal properties. The CuCrZr matrix composites reinforced with 30% and 50% volume fraction of W particles exhibited stable thermal conductivities of around 300 and 240 Wm^−1^K^−1^, respectively, over a temperature range of 300 to 600 °C [71]. The coefficient of thermal expansion of those composites was also found to stabilize in the range of 13.3 to 14.4 x10^−6^ K^−1^, and 9.7 to 10.3 x10^−6^ K^−1^ with 30% and 50% volume fraction W particle reinforcements, respectively, over a temperature range of 150 to 550 °C. In another study, a composite with 60 wt.%W–40 wt.% Cu showed stable thermal conductivity (260–240 Wm^−1^K^−1^) over a temperature ranging from room temperature to 1000 °C [78].

Molybdenum (Mo) as a reinforcement has not received much research attention. Chen et al. [80] reported a thermal conductivity of 270 Wm^−1^K^−1^ with 55% volume fraction Mo/Cu matrix composite.

#### 2.1.8. Metal Oxides

Metal oxides as reinforcements have also been the least explored. Das et al. [81] used Y_2_O_3_ and WO_3_ to synthesize Y_2_W_3_O_12_ hybrid powders. The thermal conductivities of all the composites with 40% to 70% volume fraction Y_2_W_3_O_12_ reinforcements were below 300 Wm^−1^K^−1^.

**Figure 5:**
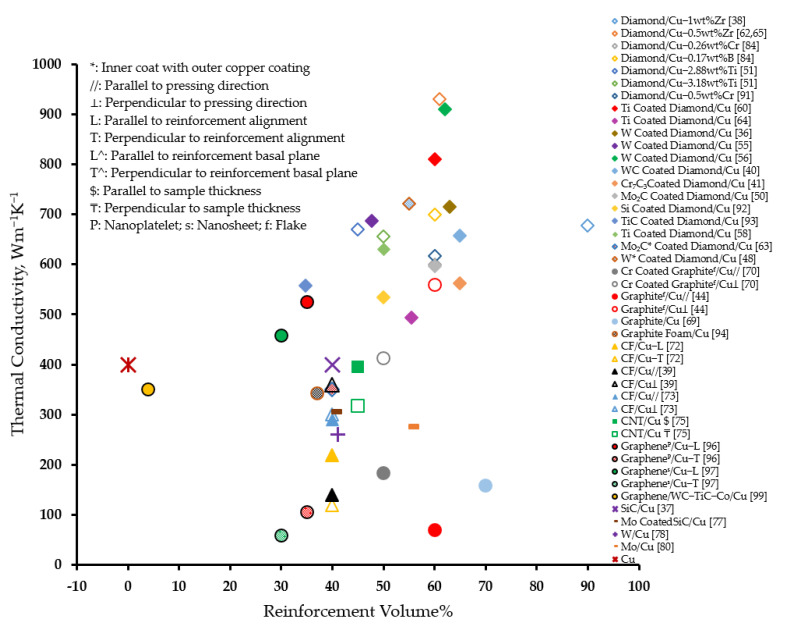
Effect of reinforcement on the thermal conductivity of copper matrix composites.

Figure 5 presents an overview of the effectiveness of various reinforcements in improving the thermal conductivity of copper matrix composites. It can be observed that, in general, diamond particles serve as an effective reinforcement to significantly improve the thermal conductivity as compared to other reinforcements. Especially, the W coating on diamond particles proved to be consistently effective in rendering high thermal conductivity to the composites as evidenced from several studies [36,40,48,55,56]. Moreover, Zr is observed to be an effective carbide-forming element for alloying copper matrix. On the contrary, a general reduction in the thermal conductivity of the composite was observed compared to that of matrix material, with the incorporation of carbon fibers, CNT, W, and Mo reinforcements. However, a minimal improvement was observed in a plane where graphene reinforcements were aligned.

**Figure 6:**
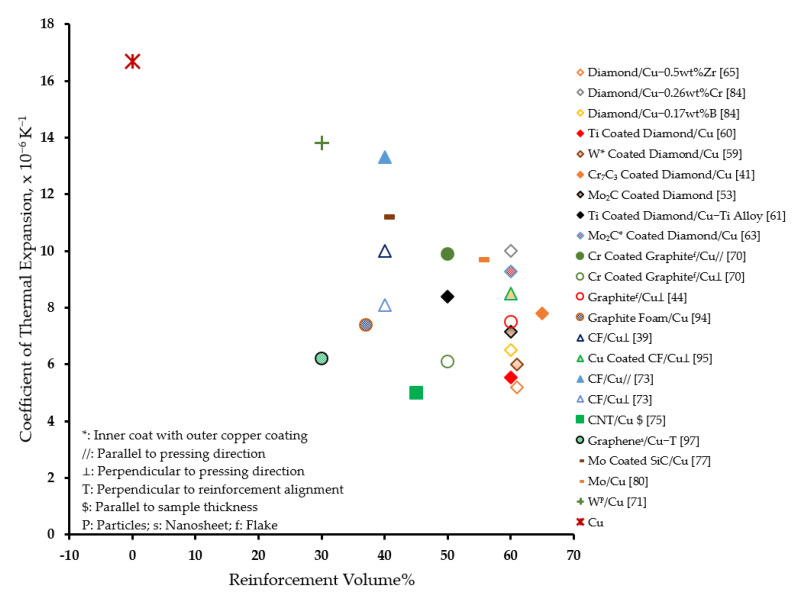
Effect of reinforcement on the coefficient of thermal expansion of copper matrix composites.

Figure 6 presents an overview of the effectiveness of various reinforcements in reducing the coefficient of thermal expansion of copper matrix composites. It can be observed that, in general, diamond particles serve as an effective reinforcement to significantly reduce the coefficient of thermal expansion as compared to other reinforcements [41,53,59,60,61,63,65,84]. The improved reinforcement–matrix interfacial bonding due to the alloying of copper matrices [65,84] and/or by the surface metallization of diamond reinforcement [41,53,59,60,63] with carbide-forming elements led to this improvement. Especially, Ti as a coating on diamond particles [60] and/or as an alloying element in copper matrix [61] proved to be effective in achieving a desired reduction in the coefficient of thermal expansion.

### 2.2. Aluminum Matrix Composites (AMCs)

Aluminum, being lighter, has a high specific thermal conductivity, which makes it the leading matrix material for heat sink composites in automotive and aerospace electronics, and also in portable electronic devices [101,102,103,104,105,106]. However, the high coefficient of thermal expansion (~23 × 10^−6^ K^−1^) of aluminum is compensated by reinforcement with carbides (SiC [33,101,107,108,109,110,111,112], B_4_C [105]), nitride (BN [107,113], Si_3_N_4_ [114], AlN [111,115]), oxides (Al_2_O_3_ [107]), diamond [102,106,116,117,118,119,120,121,122,123,124,125,126], graphite flakes [68,127,128,129], and carbon fibers [39,130,131,132,133].

#### 2.2.1. Carbide, Nitride, and Oxide reinforcements

SiC is the most commonly used carbide reinforcement for aluminum matrix composites. Saraswati et al. [111] achieved an acceptable coefficient of thermal expansion (7 × 10^−6^ K^−1^) along with a low thermal conductivity (160 Wm^−1^K^−1^). Zhang et al. [112] achieved an acceptable coefficient of thermal expansion (7.3 × 10^−6^ K^−1^) with 73 vol% SiC particle-reinforced Al-Si alloy matrix. However, the resulting AMC suffered profound thermal fatigue damage as a result of thermal cycling. Schobel et al. [33] focused on the effect of void kinetics on the development of internal stresses and the resulting macroscopic thermal expansion behavior during thermal cycling of pure aluminum and Al-Si alloy matrix reinforced with SiC particles. The thermal fatigue damage was more profound in pure aluminum matrix than in Al-Si alloy matrix. Elomari et al. [109] demonstrated that the preoxidation of SiC can enhance the thermal performance of the composite at elevated temperatures. The enhanced performance was attributed to enhancement in the volume fraction of the ceramic phase from 47 to 55% due to the formation of a silicion oxide layer on its surface. The reinforcement of Al-Si alloy matrix with bimodal SiC particles resulted in enhanced thermal conductivity of the composite [110]. The increment in the volume fraction of finer particles reduces the micro-pores and sintering time. This favors the formation of new phases (Al_9_Si and Al_3_._21_ Si_0_._47_), which contributes to the increment of the thermal conductivity. The maximum thermal conductivity (235 Wm^−1^K^−1^) was reported at 45 vol% of finer SiC particles (i.e., 0 vol% larger SiC particles).

Manivannan et al. [107] developed AMCs reinforced with micron-sized 5 vol% cubic boron nitride (CBN), SiC, and Al_2_O_3_. A comparison of their thermal performance discovered that the CBN reinforcement yielded enhanced thermal conductivity, although the value remained relatively low for most applications. In another study, Manivannan et al. [113] reinforced AA (AA6061 T6) with micron-sized CBN particles. An improved thermal conductivity compared to the base alloy using a pin-fin apparatus was reported. Though CBN is considered to be one of the best conductors and is an abrasive, its potential as an efficient reinforcement to enhance the thermo-mechanical properties of AMCs has not been fully explored. The B_4_C and AlN-reinforced aluminum composites have not attracted much research focus. Tayebi et al. [105] reported the coefficient of thermal expansion to be 8 × 10^−6^ K^−1^ with Al/25%B_4_C composite. Zhang et al. [115] reported a thermal conductivity and coefficient of thermal expansion of 130 Wm^−1^K^−1^ and 11.2 x10^−6^ K^−1^ with Al/50% AlN composite.

The efforts to develop aluminum nanocomposites for heat sink application is scarce. Matli et al. [114] fabricated Al/Si_3_N_4_ nanocomposite through the powder metallurgy route with subsequent microwave sintering and hot extrusion. A coefficient of thermal expansion of 19.3 × 10^−6^ K^−1^ (reduction by 17.2%) was reported. Reddy et al. [101] fabricated Al/SiC nanocomposites through a similar route. A coefficient of thermal expansion of 19.2 × 10^−6^ K^−1^ (reduction by 17.6%) was reported. In both instances, the resulting coefficient of thermal expansion was above the acceptable range to meet market requirements.

#### 2.2.2. Carbon-Based Reinforcements

Diamond forms weak interfacial bonds with Al matrix. Alloying matrix material with a small amount of Si was found to form SiC at the matrix–diamond interface. A thermal conductivity of 375 Wm^−1^K^−1^ and a coefficient of thermal expansion of 7 × 10^−6^ K^−1^ was achieved with AlSi/diamond (60 vol%) composite through the gas pressure infiltration route (GPI), where SiC was not formed at the interface [119]. The formation of a brittle, hydrophilic interfacial phase Al_4_C_3_ instead of SiC was considered to serve as a thermal barrier. To prevent the formation of Al_4_C_3_ and to improve interfacial bonding, the diamond particles were coated with SiC [121], TiC [116], Ti [123], and W [124]. A thermal conductivity of 365 Wm^−1^K^−1^ in combination with a low coefficient of thermal expansion of 5.69 × 10^−6^ K^−1^ was reported with 60 vol% TiC-coated diamond particle reinforcement [116]. Yang et al. [123] fabricated AMC with Al-Si alloy matrix reinforced with Ti-coated diamond particles by gas pressure infiltration. The coefficient of thermal expansion was found to range between 5.07 × 10^−6^ K^−1^ and 9.27 × 10^−6^ K^−1^ with 50 vol% diamond particle reinforcement [123]. It is to be noted that the addition of a small amount of Si to Al reduces the propagation of thermal fatigue damage and helps to provide higher thermal stability in AMC [118]. Che et al. [124] reported an outstanding thermal conductivity of 620 Wm^−1^K^−1^ with gas pressure-infiltrated W-coated diamond-reinforced AMC.

**Figure 7:**
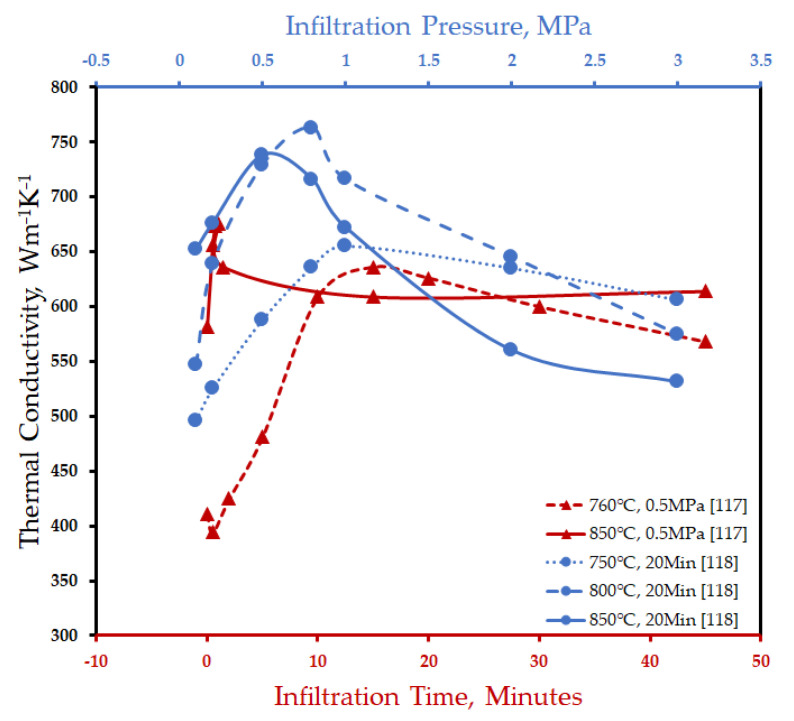
Effect of the infiltration temperature, time, and pressure on the thermal conductivity of AMCs fabricated by GPI.

**Figure 8:**
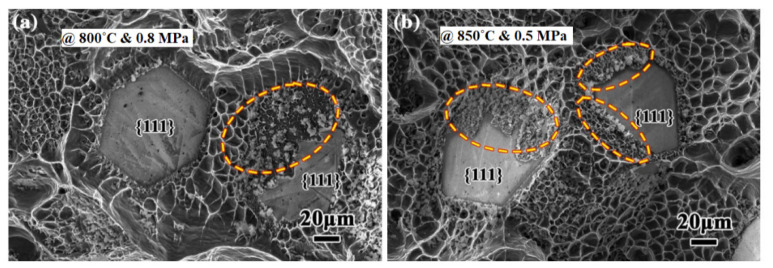
SEM micrographs demonstrating the occurrence of the diffusion reaction on the partial {111} faces of diamond particles in the gas pressure-infiltrated Al/diamond composites: (**a**) 800 °C and 0.8 MPa; (**b**) 850 °C and 0.5 MPa [126].

Significant progress was realized when Ruch et al. [102] established the superiority of the gas pressure infiltration process over squeeze casting in the fabrication of Al/diamond composite. A thermal conductivity as low as 131 W/m^−1^K^−1^ of squeeze-casted Al/diamond composite shot up to 670 W/m^−1^K^−1^ when fabricated by GPI. The characteristic long exposure time of diamond crystals to aluminum melt involved in this process promotes interfacial bonding and thermal conductance. Monje et al. [125] further explored the effect of the reinforcement-matrix melt contact time and infiltration temperature on the thermal conductivity of Al/diamond composite fabricated by the gas pressure infiltration process as presented in Figure 7. The higher the infiltration temperature, the shorter the contact time and vice-versa, which was observed to be required to attain maximum thermal conductivity. Maximum thermal conductivity of 636 Wm^−1^K^−1^ was reported at a contact time of 15 min and infiltration temperature of 760 °C. At the higher infiltration temperature of 850 °C, a maximum thermal conductivity of 676 Wm^−1^K^−1^ was achieved at a contact time of 1 min [125]. The enhanced thermal conductivity was due to the direct contact between the diamond surface and carbon-enriched Al layer (diffusion layer). The breaking away of the carbon atoms from the diamond surface and their subsequent diffusion through liquid Al results in the formation of the diffusion layer. The formation of the diffusion layer precedes the Al_4_C_3_ formation. After exceeding the solubility limit (43 atomic%) of carbon in Al, the Al_4_C_3_ phase precipitates on the diamond surface either in a particle form or as a continuous layer. For enhanced interfacial bonding and thermal conductance, the Al/diffusion layer/diamond system is preferred [125]. Zhang et al. [126] demonstrated that the process parameters can be optimized in the gas pressure infiltration process to achieve superior thermal conductivity of the Al/diamond composite by activating diffusion reaction on the diamond faces as shown in Figure 7 and Figure 8. The pressure optimization at infiltration temperatures as low as 750 °C can easily activate the diffusion reaction on the {100} face of diamond. The {111} face of diamond, being chemically more stable, requires higher energy levels to breakout carbon atoms from its surface. Therefore, to activate the diffusion reaction, this face requires temperature optimization. Moreover, higher infiltration temperatures require smaller pressure as shown in Figure 8. A thermal conductivity of 760 Wm^−1^K^−1^ was recorded at an optimum infiltration temperature and pressure of 800 °C and 0.8 MPa [126]. Later, Wang et al. [106] also demonstrated that by controlling the processing parameters, squeeze casting can be used to obtain Al/diamond composites with a thermal conductivity of 606 Wm^−1^K^−1^. The enhanced thermal conductivity was attributed to the activation of the diffusion reaction on the {111} face of diamond, resulting in good interfacial bonding and interfacial thermal conductance. Further studies are required to confirm the mechanism of improvement of thermal conductivity in Al/diamond composite and to explore the effect of Al_4_C_3_ on the thermal conductivity.

**Figure 9:**
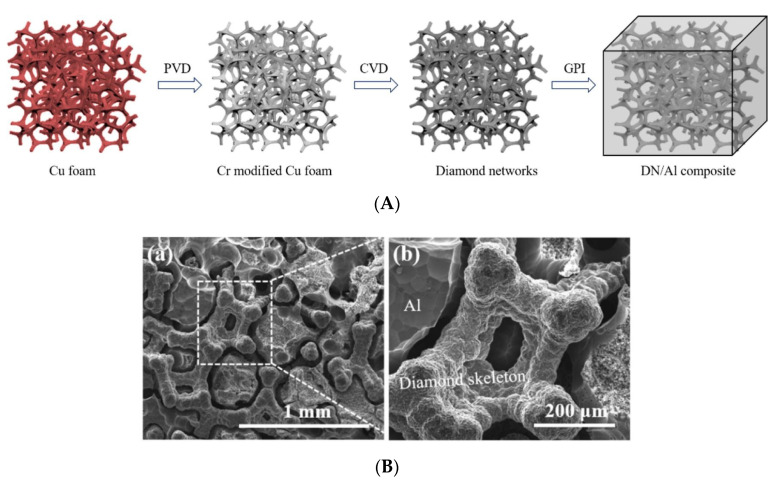
(**A**) Schematic illustration of the fabrication process of the diamond network/Al composite, (**B**) SEM morphologies of the diamond network/Al composite [134].

Some new approaches were adopted to reduce the usage of high-volume fraction of diamond reinforcement. Diamond film (6.5 vol%) was coated on spiral W wire and was reinforced in the Al matrix, resulting in a thermal conductivity of 294 Wm^−1^K^−1^ [117]. A novel technique that has been reported is to infiltrate liquid Al into diamond-coated Cr-modified copper foam as shown in Figure 9. The resulting composite featured a thermal conductivity of 315.7 Wm^−1^K^−1^ at merely 4.6 vol% of diamond [134].

Graphite flake-reinforced Al matrix composites features excellent machinability in combination with high specific thermal conductivity. Oddone et al. [129] developed 50 vol% graphite flake-reinforced AMCs. A significantly high in-plane thermal conductivity (390 Wm^−1^K^−1^) with zero or negative through-plane CTE was reported. However, a drastic reduction in hardness and tensile strength with an increasing volume fraction of graphite flakes was noticed. An increase in thermal conductivity (from 324 to 783 Wm^−1^K^−1^) was reported with an increase in the volume percentage of graphite flakes (from 10 to 80%) while the coefficient of thermal expansion reduced in the parallel (from 16.9 to −2.5 × 10^−6^ K^−1^) and perpendicular (from 15.2 to 10.1 × 10^−6^ K^−1^) direction to the basal plane [127]. Additionally, an increase in the size of the graphite flakes can increase the thermal conductivity. The maximum thermal conductivity reported was 604 Wm^−1^K^−1^ with the increase in the size of the graphite flakes from 150 to 500 µm [128]. These composites are commonly fabricated through the powder metallurgy route to avoid the formation of Al_4_C_3_, which reduces the thermal conductivity and corrosion resistance.

Another carbon-based reinforcement is carbon fiber. Beronska et al. [135] fabricated AMC by reinforcing 57.6 vol% unidirectional carbon fiber in Al-3wt% Mg alloy by the gas pressure infiltration process. Mg was added to suppress the formation of Al_4_C_3_. A thermal conductivity of 540.8 W/mK in the longitudinal direction was reported. The coefficient of thermal expansion in the longitudinal direction was found to range from −1 × 10^−6^ K^−1^ to −1.9 × 10^−6^ K^−1^ with an increase in the temperature from 100 to 300 °C. The enhanced thermal performance was attributed to the amorphous layer formed at the fiber–matrix interface in the presence of oxygen, which probably was absorbed during the infiltration. Lee et al. [136] reported a longitudinal thermal conductivity of 273.2 Wm^−1^K^−1^ with AMC reinforced with unidirectional carbon fiber fabricated by the low-pressure infiltration process. It was demonstrated that the growth rate of Al_4_C_3_ was more profound during the cooling than the infiltration process. The increase in time from 10 to 60 min to cool from 849 to 500 °C resulted in a decrease in the thermal conductivity from 273.2 to 230 Wm^−1^K^−1^, respectively. Silvain et al. [39] and Kurita et al. [131] reinforced aluminum matrix with 50 vol% carbon fibers. A small amount of Al-Si alloy (5 vol%) was added to the aluminum matrix to reduce the melting point and improve the densification of the composite upon sintering. A in-plane thermal conductivity and coefficient of thermal expansion of 258 Wm^−1^K^−1^ and 7.09 × 10^−6^ K^−1^, respectively, were reported. Tokunaga et al. [133] achieved a thermal conductivity of 323 Wm^−1^K^−1^ with 40 vol% reinforcement of carbon fibers in aluminum matrix with Al-12.2 mass% Si alloy (10 vol%). Pei et al. [132] reinforced carbon fibers into AA6063 matrix. A thermal conductivity of 407 Wm^−1^K^−1^ along the fiber direction with a very low coefficient of thermal expansion between −0.26 × 10^−6^ K^−1^ and 0.26 × 10^−6^ K^−1^ was reported.

Studies investigating the thermal performance of CNT-reinforced AMCs are scarce [130,137]. These studies focused on the contribution of CNTs to AMC based on theoretical analysis. Studies investigating the thermal performance of graphene-reinforced AMCs are also scarce. Zhang et al. [138] reported a thermal conductivity of 260 Wm^−1^K^−1^ with 0.3 wt% graphene-reinforced AMC fabricated by powder metallurgy.

Recently, hybridizing AMCs with secondary reinforcements to mitigate the side effects of adding primary reinforcements [103,139,140,141,142] and to make the resulting composite more suitable for the intended application has gained popularity. Shu et al. [103] hybridized aluminum matrix with Ti and B_4_C to form TiC_x_-TiB_2_/Al composites. The resulting thermal conductivity was just 160 Wm^−1^K^−1^. Molina et al. [139] reported a thermal conductivity of 390 Wm^−1^K^−1^ by reinforcing Al-12wt%Si alloy matrix with graphite flakes and SiC particles. Chamroune et al. [140] hybridized aluminum matrix with graphite flakes and carbon fibers, featuring an in-plane thermal conductivity of 410 Wm^−1^K^−1^ with in-plane and through plane coefficients of thermal expansion of 15 × 10^−6^ K^−1^ and 2.4 × 10^−6^ K^−1^, respectively, using solid-liquid sintering. In another study, Peng et al. [141] reinforced graphite flakes and carbon fibers in aluminum matrix, yielding a thermal conductivity of 402 Wm^−1^K^−1^. Graphite flakes were coated with copper while the carbon fibers were doped with nitrogen to improve their wettability in Al matrix and the composite was fabricated through the optimized vacuum gas pressure infiltration method. Xue et al. [142] reported a thermal conductivity of 400 Wm^−1^K^−1^ and a coefficient of thermal conductivity of 7.8 × 10^−6^ K^−1^ with diamond/SiC/ Al-7Si-0.3Mg hybrid AMC. Han et al. [143] reported a thermal conductivity of 482.14 Wm^−1^K^−1^ and a coefficient of thermal of 2.5 × 10^−6^ K^−1^ by reinforcing aluminum matrix with 70 vol% graphite flakes, and 3-D copper networks coated with 5 vol% graphene. The enhanced thermal performance was attributed to the effective heat transfer path provided by copper networks and to the better distribution of graphite reinforcements.

**Figure 10:**
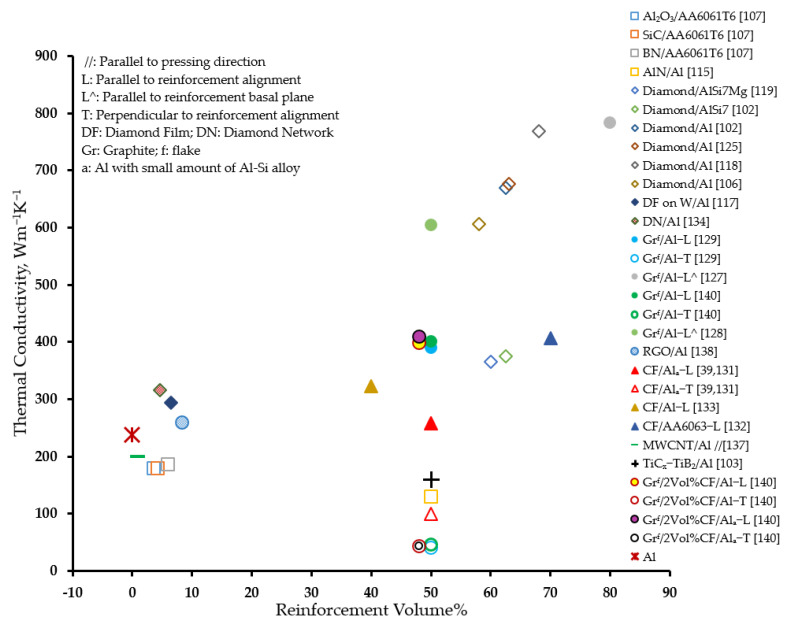
Effect of reinforcement on the thermal conductivity of AMCs.

Figure 10 presents an overview of the effectiveness of the various reinforcements in improving the thermal conductivity of aluminum matrix composites. It can be observed that, in general, diamond as well as graphite reinforcements are effective in significantly improving the thermal conductivity as compared to other reinforcements. In fact, graphite featured the highest thermal conductivity in the direction parallel to its basal plane. However, a heavy reinforcement loading in the range of 48 vol% to 80 vol% for graphite, and 58 vol% to 68 vol% for diamond was used to realize the aforementioned effect. Interestingly, a significantly high ratio of thermal conductivity to diamond loading was observed when a much smaller volume percentage of diamond was used in the fabrication of diamond network and diamond film-coated tungsten-reinforced AMCs, respectively [117,134]. On the contrary, a general reduction in the thermal conductivity of the composite was observed compared to that of the matrix material with the incorporation of Al_2_O_3_, SiC, BN, and AlN reinforcements. However, a minimal improvement was observed with reinforcement with graphene and carbon fibers.

**Figure 11:**
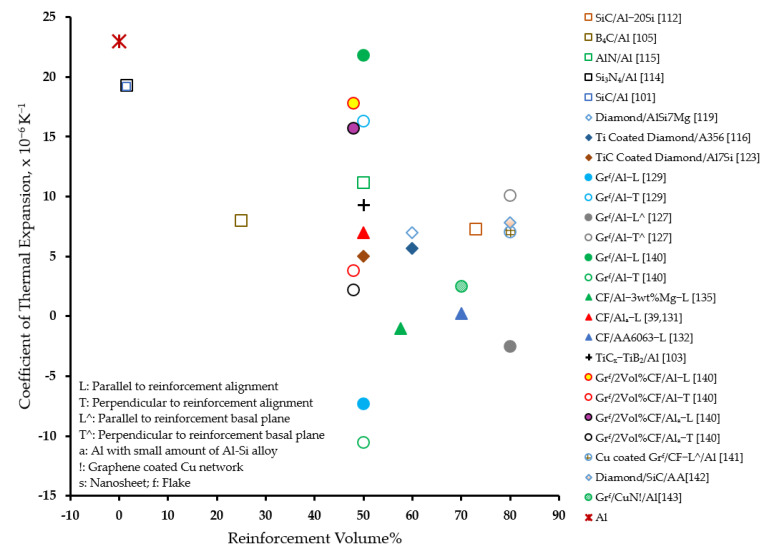
Effect of reinforcement on the coefficient of thermal expansion of AMCs.

Figure 11 presents an overview of the effectiveness of various reinforcements in reducing the coefficient of thermal expansion of aluminum matrix. It can be observed that, in general, diamond reinforcement is effective in significantly reducing the coefficient of thermal expansion as compared to other reinforcements [116,119,123]. Graphite reinforcement induced a very large anisotropy in the coefficient of thermal expansion of aluminum, ranging from a negative [127,129] to unacceptably high [129,140] values. On the other hand, hybrid AMCs present a desired reduction in the coefficient of thermal expansion [142,143].

### 2.3. Silver Matrix Composite

Silver has the highest thermal conductivity (429 Wm^−1^K^−1^) among all the matrix materials considered for thermal management. Due to its scarcity and high cost, it has been rarely investigated. Zhao et al. [144] reported a thermal conductivity of 768 Wm^−1^K^−1^ by reinforcing silver matrix with Cr-coated diamond particles. Pal et al. [145] studied the effect of functionalization of CNT reinforcement on the thermal conductivity of composite. The non-covalently functionalized CNTs resulted in a thermal conductivity of 530 Wm^−1^K^−1^ due to lower interfacial resistance.

### 2.4. Magnesium Matrix Composite

Given the low thermal conductivity (156 Wm^−1^K^−1^) of magnesium, it has also rarely been explored for thermal applications. Molina et al. [146] reported a thermal conductivity of 716 Wm^−1^K^−1^ with TiC-coated bimodal diamond particle reinforcement. The enhancement in their thermal conductivity was attributed to the nano-scale coating thickness, and to the bimodal mixture of thee reinforcing particles. Hou et al. [147] reported a low thermal conductivity of 120.6 Wm^−1^K^−1^ with Ni-coated short carbon fiber (1 vol%) reinforcement.

### 2.5. Beryllium Matrix Composite

Beryllium possesses a thermal conductivity of 200 Wm^−1^K^−1^ and a coefficient of thermal expansion of 9.5 × 10^−6^ K^−1^. Parsonage et al. [148] fabricated a beryllium matrix composite reinforced with BeO, but a marginal improvement in the thermal conductivity and coefficient of thermal expansion to 215 Wm^−1^K^−1^ and 8.7 × 10^−6^ K^−1^, respectively, was reported.

### 2.6. Indium Matrix Composite

Zeng et al. [149] reinforced indium with 50 vol% diamond particles and achieved a maximum thermal conductivity of 211 Wm^−1^K^−1^.

## 3. MMC Processing

Metal matrix composites reinforced with particles, platelets, non-continuous, and continuous fibers are essentially produced in either the liquid state or in the solid state [86,150,151]. The most popular liquid state processing methods are stir casting and liquid metal infiltration while the solid state processing route is called powder metallurgy.

### 3.1. Liquid State Processing

This method enables the incorporation of high-volume fraction of reinforcements, which is imperative to obtain a low coefficient of thermal expansion along with high thermal conductivity of the composite.

#### 3.1.1. Stir Casting Process

In this process, the reinforcements in the form of short fibers and particles are stirred into a molten metal prior to casting [22,25,81]. Manivannan et al. [107,113] used the bottom tapping stir casting method to fabricate AMCs. This method prevented oxide formation on the surface of the molten metal. The major limitation of this process is the inhomogeneous distribution of reinforcement caused by the density difference between the melt and the reinforcements [22,25,107,113].

#### 3.1.2. Liquid Metal Infiltration

##### Squeeze Casting

**Figure 12:**
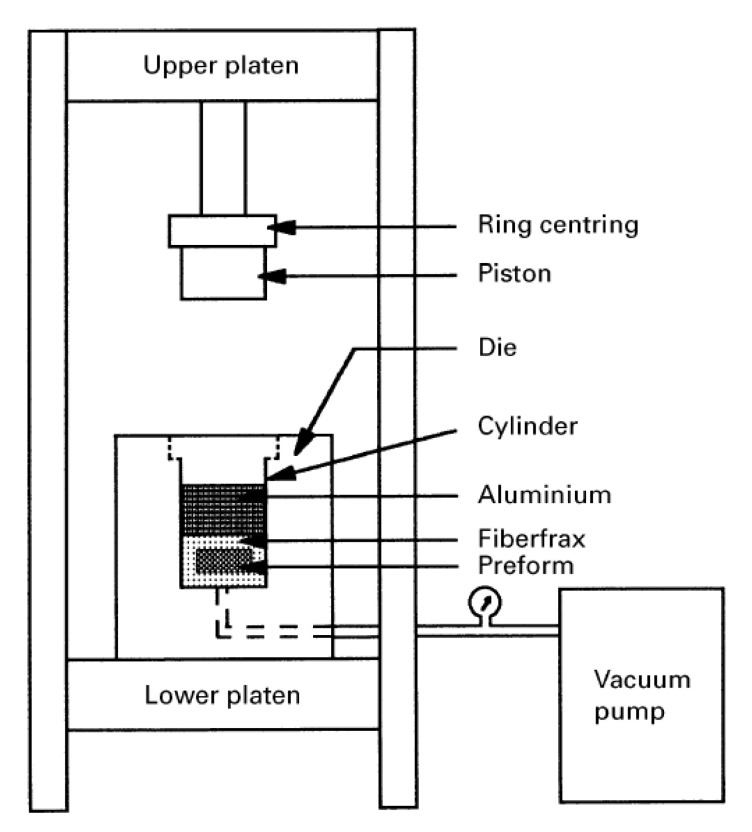
Schematic diagram of a squeeze casting unit [109].

The reinforcing particles are first pressed in a mold to make a preform. The preform is preheated in a forming gas atmosphere (94% Ar and 6% H_2_). The preheat temperature depends on the type of reinforcement. Meanwhile, the matrix metal (aluminum or copper) is melted separately. The preform is placed inside a die preheated to a relatively much lower temperature. The molten metal is immediately poured with simultaneous application of vacuum to the preform and a predetermined vertical pressure to the melt. The infiltration of molten metal into the preform is caused by the downward pressure applied by a hydraulic or pneumatic piston. The applied pressure is determined such that the piston displacement rate completes the infiltration in a few seconds before the actual commencement of solidification. The relatively cold die aids in rapid solidification of the composite under the applied pressure. The casted samples are either air cooled or annealed followed by furnace cooling to room temperature [22,25,86,150]. A schematic diagram of a typical squeeze casting unit is shown in Figure 12.

The low-temperature processing ability along with rapid infiltration and solidification have made this technique more attractive for the fabrication of AMCs [80,106,109,120,132]. The infiltration and solidification times are too short for the formation of interfacial compound (Al_4_C_3_). No graphitization of carbon fibers was observed in the fabrication of AMC by this method [132]. Neither the formation of interfacial Al_4_C_3_ nor any reaction between diamond reinforcements and Si when present in the AMC matrix were observed [102]. Rather, the addition of silicon adversely affected the coefficient of thermal expansion at high temperatures [112]. Though Khalid et al. [120] could avoid the formation of undesired Al_4_C_3_ during fabrication of AMC reinforced with monocrystalline diamond, a transformation of diamond particles into the amorphous carbon phase was reported. Pingping wang et al. [106] optimized the squeeze casting process by increasing the preheat and infiltration temperatures, and also the contact time between the diamond preform and the molten Al to promote the formation of the Al-C diffusion layer. The in situ generated thin layer diffusion layer was demonstrated to improve the interfacial bond and thermal conductivity.

##### Gas/ Vacuum Pressure Infiltration

**Figure 13:**
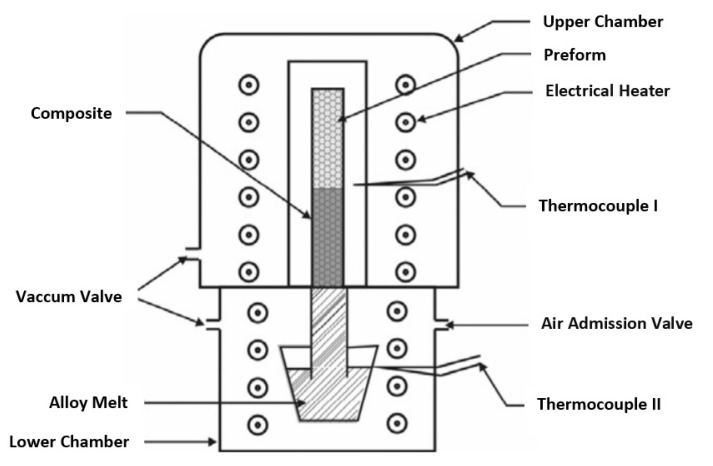
Schematic diagram of the gas pressure infiltration apparatus [142].

In this process, a pressurized inert gas or vacuum forces the molten metal/alloy in to the preform [22,86,152]. A gas pressure liquid infiltration apparatus is shown in Figure 13. The high-volume fraction reinforcement is tap packed in a steel cylinder, which is placed in the upper chamber. Aluminum/Al-alloy is placed in the lower chamber. Before melting it, the furnace chamber is evacuated to create vacuum pressure. The preform in the evacuated upper chamber is simultaneously heated to temperatures of 700–750 °C. Once the thermal equilibrium is attained, pressurized gas is allowed to enter the chambers, which forces the melt into the preform. Finally, the infiltrated sample is furnace cooled to room temperature. The preform and melt temperatures can be independently controlled using this apparatus [116,123,142].

The gas pressure infiltration technique requires a longer processing time, which has been exploited to improve the interfacial bonding between the reinforcement and metal matrix (i.e., copper or aluminum) along with matrix alloying and/or reinforcement coating with carbide-forming elements. Mostly copper matrix composites [40,41,42,43,46,47,53,60,65,66,84,91,153,154] and AMCs [118,119,134,139,155] reinforced with high-volume fraction of diamond were fabricated by this technique. Ruch et al. [102] obtained a higher thermal conductivity with the composite fabricated by the gas pressure infiltration method compared to that fabricated by squeeze casting. Formation of a thin carbide layer at the interface due to the longer exposure time was credited for the higher thermal conductivity of the processed metal.

The success of the liquid infiltration process (squeeze casting and gas pressure infiltration) depends on the porosity and the strength of the preform. The open pore structure allows the metal or alloys to flow. The strength enables the preform to withstand pressures applied during the infiltration process without becoming deformed [25,156]. Since the binder provides strength to the preform, it should be restricted to an optimum amount so that it does not block the pores.

### 3.2. Powder Metallurgy

The powder metallurgy process has emerged as an effective means for synthesizing near net shape composite products. The process not only offers a homogeneous dispersion of reinforcements in the matrix phase but can also prevent undesirable interaction between them [101,108,157]. Powder metallurgy involves milling and blending of reinforcements into metal matrix powders. The milled/blended powder mixture is further cold pressed to form a compact, which is subjected to solid state sintering. During sintering, the compact is heated to a temperature below its melting point but high enough to cause diffusion bonding. The developed microstructure determines the thermo-mechanical properties of the composite. The consolidated composite is available to be fabricated into final product through secondary thermo-mechanical processing like rolling, forging, or extrusion [22,39,73,151]. Hot press sintering, pulsed electric current sintering (PECS), and microwave sintering were reported to consolidate MMCs fabricated for heat sink applications.

#### 3.2.1. Hot Press Sintering

Hot press sintering is the conventional sintering method for MMCs. The copper matrix composites reinforced with carbon fibers (30–40 vol%) [39], SiC particles (40 vol%) [77], graphite flakes (38–60%) [44], Cr-coated graphite fibers (35–50 vol%) [70], Ti- and Cu-coated diamond particles (55 vol%) [64], and aluminum matrix composites containing B_4_C (25 vol%) [105] and graphite flakes (10–90 vol%) [127] were consolidated by hot press sintering. Besides CuCr alloy and Al containing 5–10 vol%, AlSi alloy reinforced with diamond particles (50 vol%) [44] and carbon fibers (10–50 vol%) [39,131], respectively, were also consolidated by hot press sintering. In this method, a uniaxial pressure is applied at a high temperature below the melting point [39]. The external heaters are used to heat the die and the powder contained in it. The apparently slow heating rate induces grain growth [57,108].

#### 3.2.2. Pulsed Electric Current Sintering (PECS)

In this method, the sample is heated by the applied electric field. This process involves simultaneous application of a pulsed direct current and uniaxial pressure on the powder or the compact. The current passing through the die and the compact (if conductive) is transformed into heat by a Joule heating mechanism [57,62,129,158]. A faster heating rate, shorter holding time, finer microstructure, and higher densities at lower temperatures are the advantages offered by this technique [51,57,61,129,158,159]. Spark plasma sintering (SPS) and the pulse plasma sintering (PPS) are the types of PECs. Even though the existence of plasma in SPS is contentious, it has been the most popular sintering method to consolidate MMCs. Copper matrix composites reinforced with (30–80 vol%) diamond particles were preferentially consolidated by SPS [45,54,57,61,62,63,67,90,92]. It was also used in the consolidation of CNT (0–15 vol%)-reinforced Cu [74], and Al [137] matrix composites and in carbon fiber (30 vol%) [133] and graphite flakes (50 vol%) [129] reinforced AMCs as well. The pulse plasma sintering (PPS) was used in the sintering of copper matrix composites reinforced with 50 vol% diamond particles [50,56,58].

#### 3.2.3. Microwave Sintering

This is a novel technique of sintering, which generates heat by transforming electromagnetic radiations into heat in the compact being sintered. Microwave sintering offers a high volumetric heating rate, shorter processing time, finer homogenous microstructure, improved mechanical properties, enhanced densification, reduced energy consumption, and environment friendliness over other methods [101,108,157]. Moreover, microwave sintering is cheaper and more productive than spark plasma sintering [160]. Despite all these advantages, microwave sintering has rarely been employed in research investigations involving the fabrication of MMCs intended for heat sink/thermal management application. It was used in the consolidation of 1.5 vol% SiC nanoparticle-reinforced AMC [101].

## 4. Potential Heat Sink Materials

**Figure 14:**
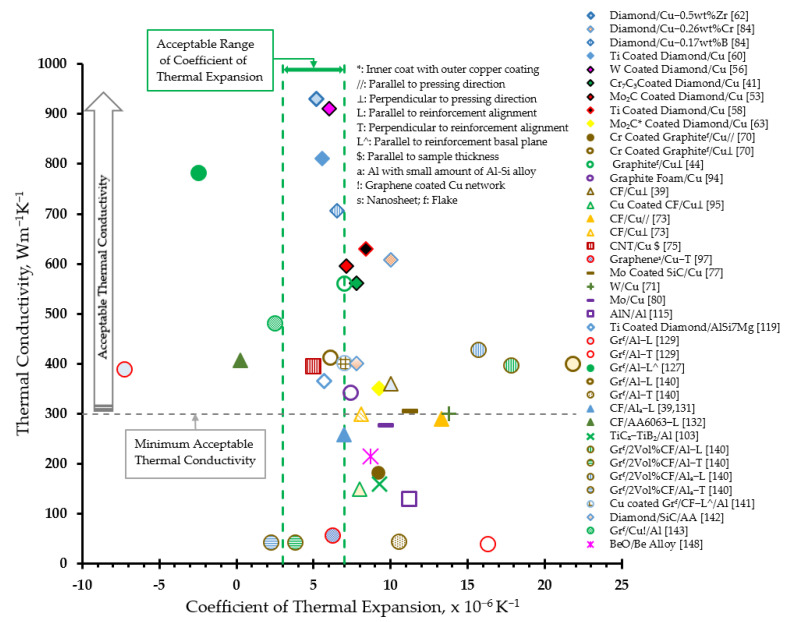
Thermal performance evaluation of metal matrix composites for heat sink applications (see Table 1 for detailed data). (Note: Color of marker outline represents a processing technique as follows: Blue- Gas Pressure Infiltration; Black-Vacuum Pressure Infiltration; Olive Green-Pressure Infiltration; Red-Spark Plasma Sintering; Yellow-Plasma Activated Sintering; Brown-Hot Press Sintering; Green-Hot Pressing; Orange-High-Temperature and High-Pressure Sintering; Purple-Squeeze Casting; Maroon-Electro Deposition).

The heat sink materials should not only have high thermal conductivities above 300 Wm^−1^K^−1^ [6,12,22], but also have coefficients of thermal expansion matching those of the semiconductor chips and ceramic substrate [6,7,11,21], typically in the range of 3 × 10^−6^ K^−1^ to 7 × 10^−6^ K^−1^ [7,11,22]. Therefore, these limits on the thermal conductivity and coefficient of thermal expansion may be referred to as an acceptable thermal performance. For novel composites to be considered as potential heatsink materials, they must demonstrate an acceptable thermal performance. Figure 14 illustrates a graphical evaluation of the thermal performance of composites in terms of the thermal conductivity and coefficient of thermal expansion. While the two vertical green lines represent the acceptable range of the coefficient of thermal expansion, a horizontal gray line represents the lower limit for the thermal conductivity.

The thermal performance of the composites can be observed to be affected not only by the type of reinforcements, but also by the techniques to improve the interfacial bonding and processing routes. The diamond particles are observed to be the most promising reinforcement in copper matrix when the interfacial bonding was improved. Alloying copper matrix by carbide-forming elements like zirconium [65] or boron [84], and surface metallization of diamond particles by carbide-forming elements like W [59] or Mo_2_C [53] are observed to be beneficial in improving interfacial bonding. Titanium-coated diamond particle reinforcement in aluminum alloy is observed to demonstrate an acceptable thermal performance [116]. Further, the gas/vacuum pressure infiltration techniques are noted to be prominent in promoting interfacial bonding due to their longer processing time.

The other carbon-based reinforcements like graphite, carbon fiber, and CNTs are also featured in Figure 14. Such reinforcements are incorporated into the matrix using the powder metallurgy route including hot pressing, hot press sintering, spark plasma sintering, high-temperature and high-pressure sintering, etc. Graphite as reinforcement in copper presents acceptable thermal properties only in the direction perpendicular to the pressing direction [44,70]. Graphite reinforcement induced a very large anisotropy in the coefficient of thermal expansion of aluminum, ranging from negative [127,129] to unacceptably high [129,140] values. An acceptable thermal performance is observed by reinforcing CNTs in copper by electrodeposition [75]. Carbon fibers have also been reinforced in copper [39,73] and aluminum [39,131,132] matrices, but the composites did not demonstrate an acceptable thermal performance. Hybridizing composites can be an effective alternative. When gas pressure infiltration was used to fabricate hybrid AMC reinforced with graphite flakes and carbon fibers, an acceptable thermal performance was demonstrated in the direction parallel to the reinforcement basal plane [141]. Graphite flake- and graphene-coated copper network-reinforced hybrid composites also feature acceptably high in-plane thermal conductivity and a coefficient of thermal expansion very close to the acceptable range [143]. Such anisotropy may seem to hinder the application of carbon-based reinforcements, although it could be successfully overcome by designing heatsinks to allow heat flow preferentially in one direction. Alternatively, graphite foam can be used to obtain isotropic properties. Graphite foam-reinforced composite is observed to demonstrate acceptable thermal conductivity and a coefficient of thermal expansion very close to the acceptable range [94].

AMCs with acceptable [122,141] and/or close to acceptable [142,143] thermal performances (see Figure 14) possess very high specific thermal conductivity in comparison to copper matrix composites with similar thermal performances. The outstanding specific thermal conductivity stems from the lower density of AMCs. This feature makes them most attractive as heat sink composites in portable electronic devices. Additionally, the AMCs containing cheaper reinforcements [141,143] offer an economical option than those containing diamond as reinforcement [122,142].

The incorporation of metal particles like Mo [80] and W [71] in copper; ceramic particles like AlN [115] and TiC [103] in aluminum; and BeO in beryllium [148] did not demonstrate an acceptable thermal performance.

## 5. Conclusions

MMCs as heat sink materials were reviewed in this paper. The heat sink materials should demonstrate an acceptable thermal performance, i.e., thermal conductivities above 300 Wm^−1^K^−1^ and coefficients of thermal expansion typically in the range of 3 × 10^−6^ K^−1^ to 7 × 10^−6^ K^−1^. The current review summarizes the efforts of different researchers in enhancing the thermal performance of metal matrix using a combination of several types of reinforcements to produce composites. Based upon this extensive literature review, the main findings can be summarized as the following:

1. Copper and aluminum are the most commonly used matrix materials for heat sink composites. Diamond particles were observed to be the most promising reinforcement when the interfacial bonding was improved either by alloying matrix or by surface metallization of diamond particles by a carbide-forming element.
2. Another important technique to improve interfacial bonding and thereby the thermal properties in Al/diamond composites is to promote the formation of an Al-C diffusion layer. The formation of a diffusion layer precedes the precipitation of a brittle, hydrophilic interfacial phase of Al_4_C_3_ that serves as a thermal barrier.
3. The MMCs with other lone carbon-based reinforcements like graphite, carbon fiber, CNTs, and graphene are generally reported to demonstrate unacceptable and anisotropic thermal performances.
4. A trend setting approach was noticed when a diamond film was coated on another reinforcement in the fabrication of AMCs. A significantly high ratio of thermal conductivity to diamond loading was observed with merely a small volume percentage of diamond. This technique needs to be further explored to establish the feasibility of this technique in providing MMCs with an acceptable thermal performance. This technique may not only reduce diamond loading but also reduce the overall cost.
5. Using graphite foam or metallic foam coated with carbon-based reinforcements is another step towards obtaining MMCs with isotropic properties. Further, it can provide an effective heat transfer path for better heat conduction.
6. The AMCs with acceptable and/or close to acceptable thermal performances possess a very high specific thermal conductivity, which makes them more attractive for heat sink applications in portable electronic devices.
7. Among the liquid state processing techniques, the gas pressure infiltration technique has emerged as being effective. This technique offers control over the infiltration temperature, pressure, and reinforcement-matrix melt contact time. This feature can be exploited to improve the interfacial bonding between the reinforcement and the matrix material.
8. The SPS and hot press sintering has emerged to be the most common sintering methods when the powder metallurgy route is adopted for the fabrication of MMCs. Though microwave sintering is hailed to offer significant advantages over other methods, it has rarely been used, and it needs to be further explored.
9. In view of the above findings, it can be concluded that the thermal performance of the composites is affected not only by the type of reinforcements, but also by the techniques to improve the interfacial bonding and processing routes. The novel techniques should be further explored to meet the ever-increasing thermal management challenges.

## Figures and Tables

**Table 1:** Metal matrix composite in Heat Sink Application: Reinforcement, Processing, and Properties.

Matrix	Reinforcement				Composite Fabrication		Thermo-Physical Properties			Ref
	Type	Size	Vol%	Pre-Treatment	Process	Parameters	Relative Density (%)	Thermal Conductivity (Wm^−1^K^−1^)	CTE (×10^−6^K^−1^)	
Cu-1 wt%Zr	Diamond	220–245 µm	90	-	HTHP	1500 °C under 5 GPa for 5 min	-	677	-	[38]
Cu-0.5 wt%Zr	Diamond	212–250 µm	61	-	GPI	1423 K under 1 MPa for 30 min	-	930	5.2	[65]
Cu-0.5 wt%Zr	Diamond	212–250 µm	-	-	GPI	1423 K under 1 MPa for 30 min	-	930	-	[46]
Cu-5 wt% B	Diamond	177–210 µm	44	-	SPS	1200 K under 30 MPa for 6 min at 150 K/min	-	660	-	[62]
Cu-0.26 wt%Cr	Diamond	200 µm	60	-	GPI	1450 K under 0.6 MPa for 30 min	-	600	10	[84]
Cu-0.17 wt%B							-	700	6–7	
Cu-2.88 wt% Ti	Diamond	30–40 µm	45	-	SPS	920–945 °C under 30–45 MPa for 10 min at 100 °C/min	97	670	-	[54]
Cu	Diamond	178–200 µm	80	Cu-0.5B-coated	SPS	1000 °C for 5 min	99.3	300	-	[45]
Cu	Diamond	150–180 µm	60	Ti-coated (220 nm)	GPI	1423 K under 1 MPa for 15 min	-	811	5.55	[60]
Cu	Diamond	75 µm	56	Ti-coated (t:285 nm)	SPS	1243 K under 40 MPa for 10 min	-	493	-	[67]
Cu	Diamond	180 µm	63	W-coated (t:220–230 nm)	VPI	1130–1150 °C under 5 Pa for 5 min	~80.2	715	-	[36]
Cu	Diamond	180 µm	61–63	W-coated (t:110–470 nm)	VPI	1130–1150 °C under 10 Pa for 5 min	~77.5–83	910–480	6	[59]
Cu	Diamond	200 µm	50	W-coated (t:260 nm)	PPS	900 °C under 80 MPa for 10 min	97	686	-	[58]
Cu	Diamond	70 µm	65	WC-coated (t:1 µm)	VPI	1150 °C under 20 Pa for 5 min	99.5	658	-	[40]
Cu	Diamond	70 µm	65	Cr_7_C_3_-coated (t:1 µm)	VPI	1150 °C under 20 Pa for 5 min	97	562	7.8	[41]
Cu	Diamond	70 µm	60	Mo_2_C-coated	VPI	1150 °C under 20 Pa for 5 min	99.5	596	7.15	[53]
Cu	Diamond	300 µm	50	Si coated (0.3 µm)	SPS	867–910 °C for 3 min under 50 MPa	96.3	535	-	[92]
Cu	Diamond	394 µm	34.7	TiC coated (300 nm)	Electrodeposition	Current: DC, 20 mA/cm^2^; pH:0.9; 150–250 rpm, 50 °C	-	557	-	[93]
Cu-0.5 wt% Ti	Diamond	180 µm	50	Ti-coated (t:0.5 µm)	SPS	1000 °C for 10 min under 50 MPa	99	630	8.4	[61]
Cu-0.5 wt%Cr	Diamond	150–180 µm	60	-	GPI	1423 K under 1 MPa for 30 min	-	617	-	[91]
				Cr-Coated			-	810	-	
Cu	Diamond	100 µm	60	Cu(outer)-W(inner) Coated	Cold Pressing+ Furnace sintering	1.2 GPa & 1100 °C for 1 h	>99.5	661	-	[48]
Cu	Diamond	400 µm	55	Cu(outer)-W(inner) Coated (310 nm)	Powder Metallurgy—HPS	900 °C under 80 MPa for 30 min	-	721	-	[64]
Cu	Diamond	10 µm	60	Cu (outer)-Mo_2_C (inner) Coated	PAS	850 °C under 20 MPa for 5 min	99.1	351	9.27	[63]
Cu	Graphite Fiber	d:10 µm; l:100–200 µm	50	Cr-coated	PM +Vacuum HPS	940 °C under 35 MPa for 40 min in vacuum (0.001 Pa)	98.04	412 ꓕ, 182 //	6.1 ꓕ, 9.9 //	[70]
Cu	Graphite Flakes	d:115 µm; t:10–20 µm	60	-	Hot Pressing	-	-	560 ꓕ, 70 //	7–8 ꓕ	[44]
Cu	Graphite Particles	30–150 µm	70	W-coated	Vacuum HPS	950 °C under 40 MPa for 30 min	~62	158	-	[69]
Cu	Graphite Foam	Cell size:300 µm	36.9	Refractory metal coating	Liquid Metal Infiltration	NA	75.2	342	7.4	[94]
Cu	Carbon-Fiber	-	40	Cu-coated	Diffusion Bonding	600 °C under 100 MPa for 15 min in vacuum	-	220 L, 120 T	-	[72]
Cu	Carbon-Fiber	d:9–10 µm; l:100–300 µm	40	-	HPS	650 °C under 50 MPa for 20 min in vacuum (0.66 Pa)	97	360 ꓕ, 140 //	10 ꓕ	[39]
Cu	Carbon-Fiber	-	60	Cu-coated	Hot Pressing	-	~69	150 ꓕ, 50 //	8–9 ꓕ	[95]
Cu	Carbon-Fiber	d:10 µm; l:100–300 µm	40	-	Hydrothermal Sintering	265 °C under 250 MPa for 60 min	100	300 ꓕ, 290 //	8.1 ꓕ, 13.3//	[73]
Cu	CNTs	d:3 nm; l:500 µm	45	-	Electrodeposition	-	-	395 $, 317 ₸	5	[75]
Cu	GO	-	1.18	-	Hot Press Sintering	900 °C under 25 MPa for 60 min	-	395	-	[98]
Cu	Graphene Nanosheets	l:5–30 µm t:5–10 nm	30	-	Vacuum Filtration + SPS	760 °C under 50 MPa for 5 min	-	458 L, 58 T	6.2 T	[97]
Cu	Graphene Nanoplatelets	l:25 µm t:12 nm	35	-	Vacuum Filtration + SPS	750 °C under 50 MPa for 5 min	-	525 L, 106 T	-	[96]
Cu	Graphene Nanosheets	t:2–10 nm	4	Cu-coated	Compaction + Furnace Sintering	900 MPa; 1000 °C, 140 min	89	350	-	[99]
	WC-TiC-Co	d:0.5–1 µm	6.71	Cu-coated						
Cu alloy (C10200)	SiC	710 µm	40	-	Stir Casting	1090 °C	-	400	-	[37]
Cu	SiC	150–210 µm	40	Mo Coated	HPS	-	-	306	11.2	[77]
Cu	W wire	-	20	-	Pressure Infiltration	-	-	300	-	[71]
Cu	W particles	-	30	-	Pressure Infiltration	-	-	~300	13.3–14.4	[71]
Cu	W particles	-	60	-	Pressure Infiltration	Infiltration of the preform at 1150 °C for 2 h	-	260–240 (RT-800 °C)	-	[78]
Cu	Mo	3.5 µm	55	-	Squeeze Casting	Infiltration of Mo preform at 30 MPa at 900–1100 °C for 5 min	-	276.2	9.7	[80]
Al-Si20 Alloy	SiC	20 µm + 60 µm (4:1)	73	-	Squeeze Casting	Infiltration of SiC preform at 100 MPa	-	-	7.3	[112]
AA356	SiC	37 µm	45	-	Compaction + Furnace Sintering	450 MPa; 500 °C, 6 h	-	235	-	[110]
AA6061T6	Al_2_O_3_	-	5	-	Stir Casting	Stirring:500 rpm for 12 min at 600 °C in Argon atmosphere; Pouring temperature: 900 °C	-	180	-	[107]
	SiC	-	5	-			-	179	-	
	CBN	-	5	-			-	186	-	
Al	B_4_C	50 µm	25	-	Hot Pressing	450 °C under 400 MPa for 1 h	-	-	8	[105]
Al	AlN	4 µm	50		Squeeze Casting	-	-	130	11.2	[115]
Al	Si_3_N_4_	15–30 nm	1.5	-	Mechanical Alloying +Microwave Sintering	500 °C	-	-	19.3	[114]
Al	SiC	15 nm	1.5	-	PM +Microwave Sintering	550 °C	-	-	19.2	[101]
Al-Si7 Alloy	Diamond	91–106 µm	60	-	GPI	750 °C under 8 MPa for 20 min	-	375	7	[119]
A356	Diamond	91–106 µm	60	Ti coated	GPI	4 KPa for 20 min	-	365	5.69	[116]
Al-Si7 Alloy	Diamond	54 µm	50	TiC coated	GPI	700 °C under 1.2 MPa	-	-	5–8.29	[123]
Al	Diamond	150–180 µm	-	W coated (45 nm)	GPI	800 °C under 1 MPa for 60 min	-	620	-	[124]
AlSi7 Alloy	Diamond	91–106 µm	60–65	-	GPI	750 °C under 8 MPa for 20 min	-	375	-	[102]
Al							-	670	-	
Al	Diamond	395 µm	62	-	GPI	760 °C under 0.5 MPa for 15 min	-	636	-	[125]
						850 °C under 0.5 MPa for 1 min	-	676	-	
Al	Diamond	150–178 µm	68	-	GPI	750 °C under 1 MPa for 20 min	99.2	655	-	[126]
						800 °C under 0.8 MPa for 20 min	99.28	760	-	
						850 °C under 0.5 MPa for 20 min	99.35	738	-	
Al	Diamond	90–106 µm	58		Squeeze Casting	800 °C under 15 MPa for 15 min	-	321	-	[106]
				-	Squeeze Casting (Optimized)	850 °C under 15 MPa for 90 min	-	606	-	
Al	Diamond	10–15 µm	4.6	-	GPI	800 °C under 5 MPa for 10 min	-	315.7	-	[134]
Al2024	Graphite flakes	d:500 µm; t:10 µm	50	-	SPS	600 °C for 10 min under 45 and 50 MPa	-	390 L; 40 T	−7.3 L; 16.3 T	[129]
Al	Graphite flakes	d:550 µm; t:10–30 µm	80	-	Vacuum Hot Pressing	913 K and 60 MPa for 1 h under 2.7 Pa vacuum	-	783 L^	−2.5 L^, 10.1 T^	[127]
Al	Graphite flakes	l:550 µm; t:30 µm	50	-	HPS	600 °C under 60 MPa for 30 min	97.5	400 L, 45 T	21.8 L, −10.5 T	[140]
Al	Graphite flakes	l:500 µm	50	-	Powder Metallurgy	-	99.6	604 L^	-	[128]
Al	RGO	-	3	-	Compaction + Sintering	200 MPa and 600 °C for 5 h in Argon	42	260	-	[138]
Al-3 wt%Mg	Carbon-Fiber	d:10 µm	57.6	-	GPI	750 °C under 5 MPa for 2 min	-	540 L	−1 to −1.9 L	[135]
Al	Carbon-Fiber	d:11 µm	-	-	Pressure Infiltration	1073 K under 0.8 MPa for 1 min; cooling-10 min	-	273.2 L	-	[136]
Al+ (5 Vol%) Al-Si alloy	Carbon-Fiber	d:8 µm; l:200 µm	50	-	HPS	600 °C under 60 MPa for 30 min	-	258 L	7.09 L	[39]
Al+ (5 Vol%) Al-Si alloy	Carbon-Fiber	d:8 µm; l:200 µm	50	-	Hot Pressing	600 °C under 60 MPa for 30 min	97	258 L	7 L	[131]
Al+ (10 Vol%) Al-Si alloy	Carbon-Fiber	d:10 µm; l:270 µm	40	-	SPS	873 K under 10 MPa for 60 min at 20 K/min	99.4	323 L	-	[133]
AA6063	Carbon-Fiber	d:10 µm; l:270 µm	67.9–70	-	Pressure Infiltration	900 °C under 5 MPa for 10 min	-	407 L	(−0.26 to +0.26) L	[132]
Al	MWCNTs	d:20–30 nm; l:10–30 µm	0.8	-	SPS	853 K under 40 MPa for 10 min at 50 K/min	-	199 //	-	[137]
Al	TiC_x_:TiB_2_	-	50	-	Hot Pressing	-	-	160	9.3	[103]
Al-12Si Alloy	Graphite flakes	d:400 µm; t:50 µm	69	-	GPI	700 °C under 2.5 MPa for 2 min	-	390 L	-	[139]
	SiCp	22.5 µm	16	-						
Al	Graphite flakes	l:550 µm; t:30 µm	48	-	HPS	600 °C under 60 MPa for 30 min	-	398 L,44 T	17.8 L, 3.8 T	[140]
	Carbon-Fiber	d:10 µm; l:250 µm	2							
Al + (10 Vol%) Al-12Si Alloy	Graphite flakes	l:550 µm; t:30 µm	48				97.9	429 L, 44 T	15.7 L, 2.2 T	[140]
	Carbon-Fiber	d:10 µm; l:250 µm	2							
Al	Graphite flakes	d:500 µm	80	Cu coated	Vacuum GPI	750 °C under 2 MPa for 5 min	95.6	402 L^	7 L^	[141]
	Carbon Fibers	d:14–16 µm; l:80–100 µm	-	N doped						
Al-7Si-0.3Mg Alloy	Diamond	d:350 µm	80	Ti coated (2 µm)	GPI	750 °C for 1 min	-	400	7.8	[142]
	SiCp	d:45 µm	-							
Al	Graphite Flakes	270 µm	70	-	Hot Pressing	660 °C under 2.5 MPa for 30 min	-	482.14	2.5	[143]
	3D Cu network	-	-	Graphene coated (5 Vol%)						
Ag-11at%Si Alloy	Diamond	200 µm	60		-	-	-	782	-	[144]
Ag	CNT	d:8 nm; l:20 µm	6	Non-Covalently Functionalized	Compaction and Furnace Sintering	320 MPa; 800 °C	91.76	530	-	[145]
Mg	Diamond	400 µm + 58 µm (70:30-Bimodal)	76	TiC coated	GPI	740 °C under 1.5 MPa for 2 min	-	716	-	[146]
E-20Be Alloy	BeO	-	20–60		-	-	-	215	8.7	[148]

l: Length; d: Diameter; t: Thickness; GPI: Gas Pressure Infiltration; VPI: Vacuum Pressure Infiltration; SPS: Spark Plasma Sintering; PPS: Pulse Plasma Sintering; PAS: Plasma Activated Sintering; HPS: Hot Press Sintering, HTHP—High Temperature and High Pressure Sintering; //: Parallel to pressing Direction; L: Parallel to reinforcement alignment; L^: Parallel to reinforcement basal plane; $: Parallel to sample thickness; ⊥: Perpendicular to pressing Direction; T: Perpendicular to reinforcement alignment; T^: Perpendicular to reinforcement basal plane; ₸: Perpendicular to sample thickness.
